# Applicability of the Nordic Occupational Skin Questionnaire for Screening Contact Dermatological Disorders in Sea Fishers

**DOI:** 10.3390/ijerph15020381

**Published:** 2018-02-23

**Authors:** Piero Lovreglio, Rossana Rotondi, Patrizia Chiarappa, Paolo Romita, Ignazio Drago, Fabrizio Guarneri, Antonella Basso, Leonardo Soleo, Caterina Foti

**Affiliations:** 1 Interdisciplinary Department of Medicine, Section of Occupational Medicine “E.C. Vigliani”, University of Bari, 701241 Bari, Italy; rossanarotondi@libero.it (R.R.); patrizia.chiarappa@uclouvain.be (P.C.); ignazio.drago@gmail.com (I.D.); antonella.basso@uniba.it (A.B.); leonardo.soleo@uniba.it (L.S.); 2 Department of Biomedical Science and Human Oncology, Section of Dermatology, University of Bari, 701241 Bari, Italy; romitapaolo@gmail.com (P.R.); caterina.foti@uniba.it (C.F.); 3 Department of Clinical and Experimental Medicine, Section of Dermatology, University of Messina, 98122 Messina, Italy; f.guarneri@tiscali.it

**Keywords:** NOSQ, contact dermatological disorders, fishermen

## Abstract

This survey aimed to evaluate the applicability of the Nordic Occupational Skin Questionnaire (NOSQ) as a preliminary screening tool to investigate the presence of contact dermatological disorders in sea fishermen. The Italian version of the NOSQ was administered to 143 male fishermen working at an Apulia (Southern Italy) Fisheries, and 136 male workers who had never worked as sea fishers (controls). A significantly higher rate of frequency of transient itchy wheals on the hands, wrists, and forearms was recorded in the fishermen as compared to the controls (49.6% vs. 8.1%; *p* < 0.001), while there was no significant difference in the frequency of eczema (8.4% vs. 6.6%). In 46.1% of the fishermen, the onset of transient itchy wheals was associated with contact with specific agents and the most common causes were algae and aquatic plants (49.3%) and seabed sludge (25.3%). In conclusion, the administration of the NOSQ can be useful in preliminary screening for dermatitis in fishermen, although it could show a possible overestimation of the prevalence of transient itchy wheals.

## 1. Introduction

Occupational skin disorders are among the most common occupational diseases worldwide, and are often caused by contact with specific agents present in workplaces. In some occupational categories—such as hairdressers, health care workers, metalworkers, cleaners, and workers in the food and construction industry—contact skin disorders have been amply reported [1,2,3,4]. Instead, there are few reports in literature about the prevalence of these disorders in workers in the fishing sector, even if this occupation is very widespread in many nations with long coastlines, lakes, and rivers, and involves exposure to many noxae that can cause the onset of dermatitis. In fact, possible causes are acute or chronic contact not only with fish and seafood products, but also with various agents present as contaminants, such as algae, bacterial or marine toxins, gas produced by the decomposition of fishing products in anaerobic conditions, as well as chemical additives used as preservatives [5].

To evaluate the prevalence of occupational contact dermatitis, as well as in the health surveillance of the onset of such forms, it would be useful to be able to rely on simple tools for preliminary data collection both at individual and at occupational group level. This was the rationale for the development, in 2002, of the Nordic Occupational Skin Questionnaire (NOSQ), a standardized, reproducible questionnaire applied to assess, in working populations, the presence of eczema and transient itchy wheals [6]. Although the NOSQ has now been validated in many languages, its utility for screening dermatological conditions in some worker categories as fishermen, where their prevalence and significance is unknown, has been little investigated. 

The aim of this survey was to evaluate the applicability of administering the NOSQ to sea fishermen as a preliminary assessment tool to screen for the presence of contact dermatological disorders.

## 2. Materials and Methods

### 2.1. Workers

The study included 143 male fishermen working at the Fisheries in Mola di Bari (Apulia, Southern Italy), who had worked for at least one year. The fishermen were compared with 136 male workers, matched for age classes, with technical, administrative, or teaching jobs, who had never worked as sea fishers (controls). Exclusion criteria were previous other job at high risk for the onset of dermatological diseases (hairdressers, health care workers, metalworkers, cleaners).

The fishing activity was trawling in 82.4% of the fishermen, using gillnets in 6%, and longlines in 2.8%, while 9.2% did both trawling and longline fishing. The fishing worked was within 20 miles of the coast for 74.8% of them (small fishing and local coastal fishing), while 25.2% would travel as far as 40 miles from the coast (coastal fishing). All the fishermen had contact with the catch during lowering and pulling in the fishing lines and nets, and when selecting the fish and stacking them in crates.

The fishermen and controls were administered a general questionnaire probing information about their current job, previous working activities, and lifestyle, as well as the Italian version of the long Nordic Occupational Skin Questionnaire NOSQ-2002 (NOSQ-2002/LONG) [7]. The latter is a standardized questionnaire consisting of 57 questions about any onset of signs or symptoms of dermatological problems like eczema and transient itchy wheals (appearing and disappearing suddenly within hours). Particular attention was paid to any possible influence of the working activity on the appearance of the disorders.

After a full explanation of the methods and aims of the study, all participants gave written informed consent to take part. The research was conducted in accordance with the Helsinki Declaration; it was consistent with ethical public health practice, and complied with regulatory and statutory mandates to maintain, protect, and restore community health.

### 2.2. Statistical Analyses

Statistical analyses were done with software SPSS (version 14.0, Chicago, IL, USA). For continuous variables, values were expressed as means and medians and for discrete variables as frequencies. Statistical comparisons were made for continuous variables using parametric tests for comparison of the means, after verifying a normal distribution with the Kolmogorov-Smirnov test. For discrete variables the Chi square test was applied. The level of significance was set at *p* < 0.05.

## 3. Results

Age and alcohol consumption were not different between the groups, whereas the fishermen had a higher Body Mass Index (BMI) and more of them were smokers than in the control group. The years of work as fishers ranged between 1 and 53 (Table 1).

Some characteristics of the working activities were investigated by the NOSQ (Table 2). Current or previous use of gloves during working activities was significantly higher in the controls as compared to the fishermen (*p* < 0.001), particularly the use of latex/natural rubber gloves (74.3% vs. 45%). Fishermen reported a significantly higher frequency of wet work (*p* < 0.001) and of the number of times they washed their hands per working day (*p* < 0.001).

Symptoms of nasal (4.2% vs. 14.0%) and eye (7.7% vs. 25.7%) allergy were significantly lower in the fishermen than in the controls, while no significant difference was observed for asthma (Table 3). The frequency of dry skin was also significantly lower in the fishermen than in the controls (12.0% vs. 27.9%), while a significantly higher rate of frequency of transient itchy wheals on the hands, wrists, and forearms was recorded in the fishermen as compared to the controls (*p* < 0.001), considering both the life prevalence (49.6% vs. 8.1%) and the incidence of new episodes in the last year (28.7% vs. 2.2%). Instead, there was no significant difference in the frequency of eczema (8.4% vs. 6.6%) (Table 3). The age of onset of eczema was before 18 years in three fishermen, in one case between 6 and 14 years and in two cases between 14 and 18 years, versus one subject of the control group, between 14 and 18 years. No relation was found in the fishermen between the prevalence of transient itchy wheals or eczema, and age, lifestyle, years of work and type of fishing activity carried out, use and types of gloves, and number of times they washed their hands per working day (data not shown).

In 46.1% of the fishermen, the onset of transient itchy wheals was associated with contact with specific agents, while only 3.5% referred that the onset of the disorder was definitely not caused by contact with any agents. The most common cause was contact with algae and aquatic plants (49.3%), followed by seabed sludge (25.3%) (Table 4). In the controls, only 4.4% referred that the onset of itchy wheals was associated with contact with specific agents, versus 3.7% who declared that they had nothing to do with contact with such agents, a percentage comparable to the one recorded in the fishermen. Wearing latex/natural rubber gloves was reported to be the cause of the onset of skin symptoms by two fishermen and three controls, two of which referred the contact with rubber gloves as the cause of onset of itchy wheals. In all the cases, except one control, after the onset of skin symptoms, they changed the type of gloves used. Other causes of the onset of itchy wheals referred by individual control subjects were the contact with detergents, animal fur, grass/bushes, metal objects.

## 4. Discussion

Application of the NOSQ in sea fishermen demonstrated a greater prevalence of episodes of transient itchy wheals, but not of eczema, in these workers as compared to the controls.

Use of the NOSQ makes it possible to carry out a preliminary investigation of some contact dermatological disorders on the basis of the medical history, but not to make a specific diagnosis of these diseases. In our study, the NOSQ demonstrated a high prevalence of transient itchy wheals in the fishermen that could be symptoms of contact urticaria, a disease that may have either an allergic or a non-allergic etiology. To make a definitive diagnosis of allergic forms, a skin prick test with immediate reading must be performed, using commercially available protein products or else, in view of the heterogeneity of the possible causes—prick by prick—using fresh products [8,9].

Considering the results obtained, and the high percentage of controls who referred to the onset of transient itchy wheals as compared to the expected prevalence of urticaria in the general population with no occupational exposure to specific causes, the NOSQ seems to have a tendency to induce an overestimation of the presence of these lesions [10]. Differences in the translation of the questionnaire and/or the explanations given during its administration could play a role in determining this overestimation. Considering the findings in the controls, we may hypothesize an overestimation of the frequency of transient itchy wheals also in fishermen. Thus, the interpretation of the results of the NOSQ as a surveillance tool in this category of workers must be treated with some care. In any case, it seems to be essential to complete the examinations using further diagnostic tools in subjects referring the onset of itchy wheals, also in order to make a clear identification of the causal agents of such disorders in sea fishers.

Instead, the frequency of eczema seems to be largely in line with reports in literature, and no underestimation of the prevalence of this disorder, already described in previous studies, was observed [11].

In the available literature, no studies have previously identified fishing as a working activity at major risk of the onset of dermatitis, despite it being well-known and documented that there is a connection between the role of contact when eating or handling fish and seafood products in the onset of dermatitis, especially contact urticaria. In fact, there have been a number of case reports of occupational urticaria due to contact with fish and shellfish among workers in the fisheries industry packaging or preparing fish products [12,13,14]. Moreover, Harvima R.J. et al. identified the histamine present as a contaminant in fish and crabs as the causal agent of contact urticaria in a female worker who handled and fed fish and crabs at work [15]. However, in our study, the cause of onset of transient itchy wheals most frequently referred by the fishermen was skin contact with algae, aquatic plants and seabed sludge, whereas handling the catch seemed to play a secondary causal role. This may be explained by the different types of contact with fish products in different working activities [16].

Unexpectedly, the characteristics of the fishermen's activities did not seem to influence the onset of transient itchy wheals, particularly the type of fishing activities and the number of times they washed their hands per working day, although the results confirmed that it can be considered a wet work. Moreover, the questionnaire results did not show a clear causal or protective role of the use of gloves during the working activities.

## 5. Conclusions

To our knowledge, this survey reports the first experience of employment of the NOSQ to study the prevalence of some occupational contact dermatological disorders in a population of sea fishermen. The results obtained in the control group seem to indicate a possible overestimation of the prevalence of transient itchy wheals, and so pose some limitations to be taken into account also when interpreting the results obtained in the fishermen. Nevertheless, the administration of the NOSQ can be useful in preliminary screening for dermatitis in this category of workers. It also demonstrates the importance of adopting adequate preventive measures to prevent contact in fishermen not only with the catch, but also with other factors like algae, aquatic plants, and seabed sludge, that have not previously been taken into much consideration as possible causal agents of urticaria.

## Figures and Tables

**Table 1:** General characteristics, years of work, and lifestyle in the fishermen and controls.

Characteristic	Fishermen (*N* = 143)				Controls (*N* = 136)			
	*N*	Mean ± SD	Median	Range	*N*	Mean ± SD	Median	Range
Age	143	43.8 ± 11.0	43.8	20.7–69.3	136	45.1 ± 11.1	45.4	23.7–68.9
BMI ^a^	143	28.2 ± 5.1	27.5	18.1–48.3	136	26.2 ± 3.3	25.8	18.0–34.4
Years of work	143	22.8 ± 11.0	23.0	1.0–53.0	136	15.2 ± 10.9	13.0	1.0–45.0
**Years of work classes**								
<10 years	17 (11.9%)				53 (39.0%)			
10–25 years	69 (48.2%)				56 (41.2%)			
26–40 years	50 (35.0%)				26 (19.1%)			
>40 years	7 (4.9%)				1 (0.8%)			
**Smoking habit ^a^**								
Smoker	83 (58.0%)				31 (22.8%)			
Non-smoker	27 (18.9%)				68 (50.0%)			
Ex-smoker	33 (23.1%)				37 (27.2%)			
**Alcohol consumption**								
Teetotal	10 (7.0%)				13 (9.5%)			
Habitual	98 (68.5%)				90 (66.2%)			
Occasional	35 (24.5%)				33 (24.3%)			

^a^ *p* < 0.001.

**Table 2:** Some characteristics of the working activities in the fishermen and controls.

Working Characteristics	Fishermen (*N* = 143) *N* (%)	Controls (*N* = 136) *N* (%)
**Gloves use current or past ^a^**		
No	42 (29.4%)	21 (15.4%)
Only late	50 (34.5%)	64 (47.1%)
Latex and other types	15 (10.5%)	37 (27.2%)
Only other types	36 (25.2%)	14 (10.3%)
**Hours of wet work per working day ^a^**		
None	0 (0.0%)	117 (87.5%)
Less than half an hour	4 (2.8%)	9 (6.6%)
Half an hour–two hours	3 (2.1%)	4 (2.9%)
More than two hours	136 (95.1%)	4 (2.9%)
**Number of times they washed their hands per working day ^a^**		
1	14 (9.8%)	43 (31.6%)
2	41 (28.7%)	63 (46.3%)
3	35 (24.5%)	21 (15.5%)
4	53 (37.0%)	9 (6.6%)

^a^ *p* < 0.001.

**Table 3:** Frequency distribution of allergic symptoms, dry skin, eczema and transient itchy wheals on the hands/wrists/forearms investigated with the NOSQ in the fishermen and controls.

Allergic Symptoms and Skin Lesions on the Hands/Wrists/Forearms	Fishermen (*N* = 143) *N* (%)	Controls (*N* = 136) *N* (%)
Symptoms of nasal allergy	6 ^b^ (4.2%)	19 (14.0%)
Symptoms of eyes allergy	11 ^b^ (7.7%)	35 (25.7%)
Asthma	8 (7.0%)	16 (11.8%)
Dry skin	17 ^a^ (12.0%)	38 (27.9%)
Eczema	12 (8.4%)	9 (6.6%)
Transient itchy wheals	71 ^b^ (49.6%)	11 (8.1%)
Transient itchy wheals with new episodes in the last 12 months	41 ^b^ (28.7%)	3 (2.2%)

^a^ *p* < 0.01; ^b^ *p* < 0.001.

**Table 4:** Frequency of causes of onset of transient itchy wheals referred by the fishermen who manifested these lesions (*N* = 71).

Causal Agents *	%
Algae/aquatic plants	49.3
Seabed sludge	25.3
Fish, crustaceans, mollusks, cephalopods, jellyfish	16.9
Other work-related causes (exposure to the sun, mucilage, waxed protective overalls, bait, detergents/washing powders)	15.5
Non occupational causes (weeds, bushes, dog fur)	2.8

* 12 fishermen referred more than one cause.
